# The Metropolitan Asylum Board

**Published:** 1898-06-25

**Authors:** 


### The Institutional Workshop.

### THE METROPOLITAN ASYLUM BOARD.

The fourteenth annual report of the Metropolitan Asylums Board gives an account of what has been done in providing accommodation for certain classes of children, whose reception had previously devolved upon the guardians. With this object they have acquired sites near Brentwood, in Essex, and at Swanley, in Kent, for the erection of schools in which children suffering from diseases of the eye may receive both treatment and education; and they have also offered to take over the large district school at Sutton for the purposes of a third ophthalmic school. A ringworm school has not yet been provided, but the subject is receiving "anxious consideration." For children requiring the benefit of seaside air, the managers have determined to provide, "in the first instance," for 360 of these cases, divided between Herne Bay, Margate, and various small seaside homes containing about twenty-five children each, which have yet to be erected. For children who are physically or mentally defective, small houses are to be rented in different parts of London, in which groups of about eight will be looked after by foster-mothers, and go to the special schools provided for such cases by the London School Board. In regard to children under remand, and who have hitherto been sent to workhouses, it has been decided, tentatively, to provide for 150 cases by renting or purchasing houses within a convenient distance of the principal metropolitan police stations. Reverting now to the older and more ordinary work of the Board, we find that they possess and manage—(*a*) four imbecile asylums with accommodation for 6,097 beds; (*b*) the *Exmouth* Training Ship for 600 boys; (*c*) ten Fever Hospitals with beds for 4,553 patients; (*d*) one new Fever Hospital for 520 patients, approaching completion; (*e*) 1,492 small-pox beds, including those for convalescents; (*f*) six ambulance stations; (*g*) three piers and wharfs, together with steamers for river service. The report before us is a short record of an immensity of good work well done. Money has, no doubt, been spent, and here and there may have been spent freely, but of the benefit to London of the work done by the Asylums Board there can be neither doubt nor question,

